# Maternal obesity and the metabolic syndrome in reproductive health: assessing incretin-based interventions

**DOI:** 10.1007/s11154-026-10053-6

**Published:** 2026-05-19

**Authors:** Luisa Wallentowitz, Mariana G Garcia, Catalina Atorrasagasti, Charlotte Harms, Evelyn A Huhn, Pia Roser, Stefan Verlohren, Emma M Giesen, Sandra M Blois

**Affiliations:** 1 Department of Obstetrics and Fetal Medicine, University Medical Center Hamburg- Eppendorf, Martinistrasse 52, Hamburg, 20246 Germany; 2 Glyco-HAM, a Cooperation of Universität Hamburg, Technology Platform Mass Spectrometry and University Medical Center Hamburg-Eppendorf, Hamburg, Germany; 3 Junior Research Center for Reproduction: Sexual and Reproductive Health in Overweight and Obesity (SRHOO), Hamburg, Germany; 4 Experimental Hepatology and Gene Therapy Program, Instituto de Investigaciones en Medicina Traslacional (IIMT), CONICET - Universidad Austral, Pilar, Argentina; 5 Obesity Center, Department of Endocrinology and Diabetology, University Medical Center Hamburg-Eppendorf, Hamburg, Germany

**Keywords:** Maternal obesity, Metabolic syndrome, Reproductive health, GLP-1 receptor agonists, Incretin-based therapies, Pregnancy outcomes

## Abstract

The global epidemic of overweight and obesity threatens gynecological and reproductive health, necessitating effective therapeutic strategies to improve maternal and fetal long-term health outcomes. This review provides an overview of weight loss interventions and discusses their use in the context of gestation, such as surgical interventions and anti-obesity medications. As the interest in incretin-based therapies has risen substantially, we discuss how incretin-based therapies, including glucagon-like peptide-1 (GLP-1) receptor agonists and GLP-1 receptor agonists and glucose-dependent insulinotropic polypeptide (GIP) dual agonists, might improve reproductive function and could interact with the physiological metabolic changes ensuring a healthy pregnancy. These metabolic adaptations during pregnancy arise from the integration of insulin signaling, lipid metabolism, placental endocrine function, mitochondrial remodeling and inflammatory regulation. Furthermore, we consider not only short-term consequences of maternal overweight and obesity but also focus on fetal long-term health trajectories following pregnancy-related metabolic disorders, as maternal obesity and gestational weight gain are considered risk factors for childhood obesity and overweight.

## Introduction

The global prevalence of overweight and obesity has risen substantially. Currently, one-third of the world’s population is affected [1], with women suffering more frequently from obesity than men [2]. Obesity is classified by the World Health Organization as a chronic disease arising from multiple factors, such as the interaction between genetics, neurobiology and nutrition. It is diagnosed by calculating the body mass index (BMI), whereas in adults, a BMI of 25 or more is classified as overweight and a BMI of 30 is classified as obese [3]. Obesity, alongside glucose intolerance, hypertension, and dyslipidemia, is an essential component of the metabolic syndrome (MetS) [4]. These interrelated risk factors increase cardiovascular morbidity and mortality [5]. Moreover, metabolic disorders are increasingly common in individuals of reproductive age and represent major, but often overlooked, contributors to subfertility and adverse reproductive outcomes [6], leading to substantial consequences for gynecological health. The rising trend in non-communicable diseases (NCDs) is attributed to several factors including aging population, genetic predisposition and socio-demographic factors, such as nutrition and lifestyle [7].

In this review, we summarize how obesity and maternal metabolic maladaptation influence reproductive function, the mother’s and the offspring’s lifelong metabolic health. As the prevalence of obesity continues to rise and the use of modern obesity therapies including bariatric surgery and incretin-based agents expands, it becomes increasingly important to delineate the distinct reproductive effects of these interventions [8]. We compare established weight-loss interventions with special emphasis on bariatric surgery and the use of incretin-based therapies in reproduction as well as during and after pregnancy. Moreover, we discuss the need to derive personalised medicine approaches to evaluate who would be a high-risk candidate to benefit from using incretin-based therapies during gestation.

## Reproductive-age origins and lifelong trajectories of metabolic change

### Metabolic (Mal-)adaptations in reproductive health

Obesity poses a growing threat to reproductive health in a multifaceted way. It increases the risk of nonovulatory menstrual cycles, negatively affecting fecundity and delays the time to conception [9, 10]. Moreover, obesity impairs the female reproductive system through interference with the hypothalamic-pituitary-ovarian axis and by affecting ovarian function.

Women with obesity show altered concentrations of the pituitary gonadotropins luteinizing hormone (LH) and follicle-stimulating hormone (FSH), key regulators in follicle development and ovulation, consequently leading to menstrual irregularities and ovulatory dysfunction [11]. Hormonal imbalance, especially between estrogen and progesterone, may negatively influence the endometrium, leading to an impaired embryo implantation [11, 12]. Beyond these endocrine effects, metabolic disease is associated with chronic low-grade inflammation, oxidative stress, and lipotoxicity. These systemic disturbances compromise oocyte quality, endometrial receptivity, and early placental development, thereby reducing fertility and increasing the risk of early pregnancy loss [13, 14].

Adipokines, cytokines mainly secreted by adipocytes, regulate immune response, glucose and lipid metabolism, notably, among others, leptin and adiponectin. Their dysregulation negatively influences female reproductive function [15]. Women suffering from obesity have elevated fasting plasma levels of leptin, showing insensitivity to this anorexigenic hormone [11, 16]. Leptin receptors are expressed in human ovarian cells [17], and dysregulated leptin signaling may result in ovulatory dysfunction [18]. Adiponectin, an adipokine that may positively affect reproduction due to its anti-inflammatory effects and by maintaining energy homeostasis in the endometrium, is decreased in patients with MetS [19, 20]. Altogether, these findings indicate that reproductive dysfunction in metabolic disease arises from systemic pathophysiology rather than isolated gonadal defects, necessitating the need for interventions that target the metabolic dysregulation in women of reproductive age with obesity.

Polycystic ovarian syndrome (PCOS), the most common endocrine disorder among women of reproductive age, demonstrates the intertwined relationship between female reproduction and metabolism. The prevalence of this endocrine disorder ranges between 10% and 13% [21], and is increased among individuals with overweight and obesity, with a prevalence between 50% and 80% [22]. PCOS is defined as the presence of at least two of the following: clinical and/or biochemical hyperandrogenism, irregular menstrual cycles, polycystic ovarian morphology on ultrasound, whereas other endocrine causes, such as Cushing’s syndrome, should be excluded [21]. Signs of clinical hyperandrogenism include acne, female pattern hair loss and hirsutism [21]. The etiology of PCOS remains not entirely understood. Various factors are involved in the disease’s etiology, including insulin resistance, altered steroidogenesis, oxidative stress, genetics as well as environmental influences (lifestyle, diet) [23]. The hormonal imbalance in PCOS includes high androgen levels, driven by an increased pulsatile secretion of gonadotropin-releasing hormone and therefore and altered release of gonadotropins (higher LH and normal or lower FSH) [24, 25]. High LH stimulates ovarian theca cells to produce androgens, leading to hyperandrogenemia and the inhibition of folliculogenesis [24]. As a result, follicle maturation arrest, formation of small follicles and impaired ovulation occur [26]. Increased oxidative stress and hormonal imbalance are the main factors leading to impaired ovarian tissue and thereupon to infertility [23, 25]. Women with obesity and PCOS are more prone to insulin resistance, which further impedes the condition of the impaired follicle formation. Hyperinsulinemia lowers circulating sex hormone-binding globulin - concentrations, promoting androgen production by increasing the bioavailability of testosterone [24, 27]. The prevalence of insulin resistance in females with PCOS is 75% in women with normal weight and 95% in women with overweight [28], emphasizing a role of insulin resistance in the heterogenous pathophysiology of this endocrine disorder. Moreover, PCOS is associated with a higher risk of developing pregnancy complications, such as gestational diabetes mellitus (GDM) [29]. Women with PCOS have a more than threefold increase in MetS prevalence [30], and a 15-fold higher risk of infertility compared to women without [31].

### Metabolic (Mal-)adaptations during pregnancy in health & disease

#### Physiological adaptations and pathways during pregnancy

**Fig. 1:**
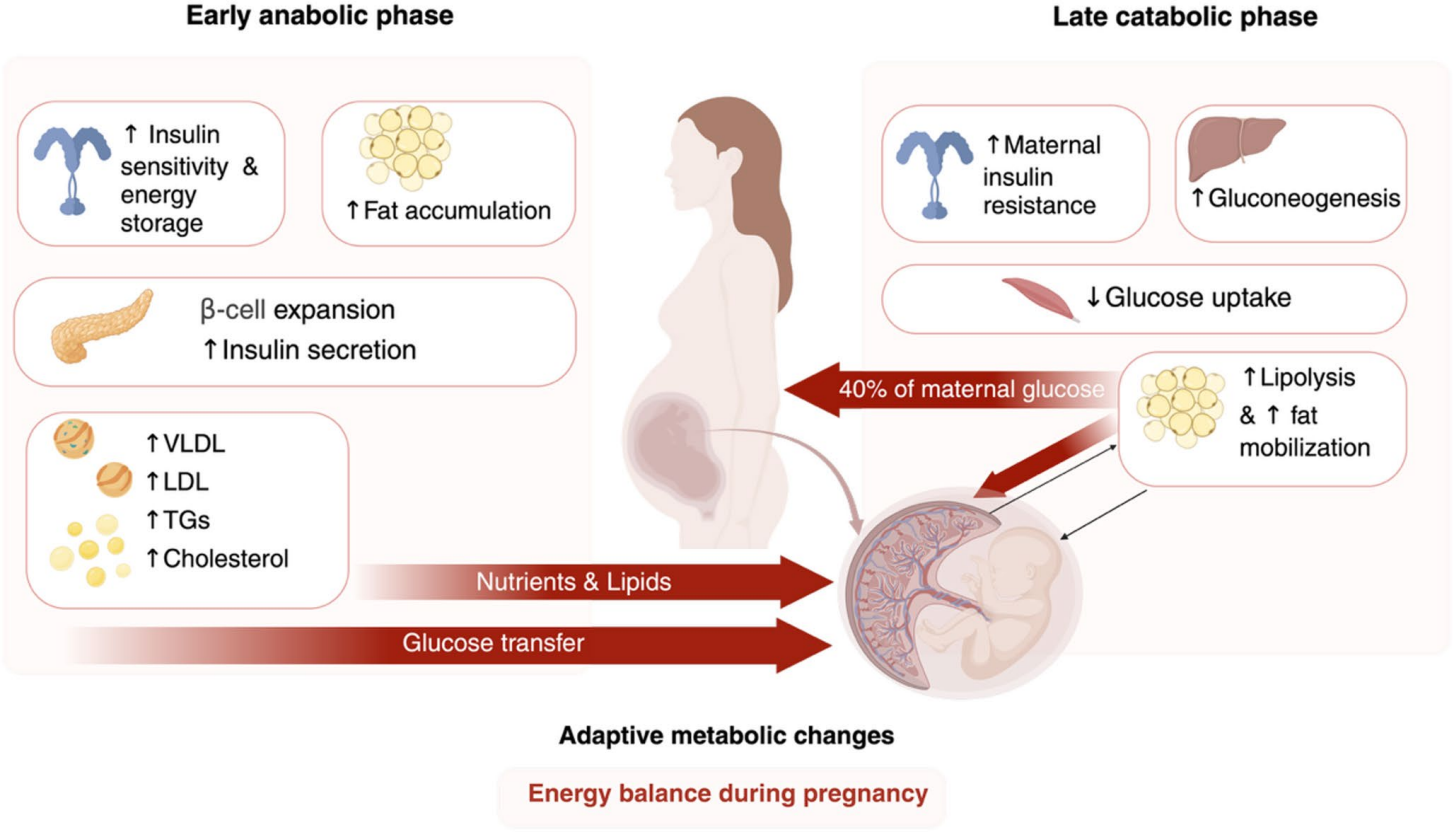
Illustrates metabolic adaptations during pregnancy, with an early anabolic phase characterized by increased insulin sensitivity and energy storage and a late catabolic phase marked by maternal insulin resistance, gluconeogenesis, reduced glucose uptake, and lipolysis to support nutrient transfer and prioritize glucose availability for the fetoplacental unit. TGs, triglycerides; LDL, low-density lipoproteins; VLDL, very low-density lipoproteins

During pregnancy, the female organism undergoes profound physical, hormonal, and humoral adaptations to ensure adequate allocation of nutritional resources for fetal development to meet increased maternal demands of gestation, delivery, and lactation (Fig. 1). This metabolic adaptation is a tightly coordinated process involving integrated changes in glucose, lipid, amino acid, and energy metabolism across maternal tissues, the placenta, and the fetus [32]. Pregnancy metabolism evolves through two phases: an early anabolic phase characterized by increased insulin sensitivity and energy storage, followed by a later catabolic phase in which stored substrates are mobilized to meet maternal, placental, and fetal demands [33]. As gestation progresses, glucose becomes the primary energy substrate for both the placenta and fetus, with the placenta consuming approximately 40% of maternal glucose. This high demand promotes progressive peripheral insulin resistance in mid-to-late pregnancy, accompanied by increased hepatic gluconeogenesis and reduced glucose uptake in skeletal muscle and adipose tissue. Normoglycemia is maintained through adaptive pancreatic responses, including expansion of β-cell mass and increased insulin secretion [34]. Also, pregnancy induces a profound reprogramming of lipid metabolism. Early pregnancy insulin sensitivity is associated with elevated circulating triglycerides, total cholesterol, low-density lipoproteins, and very low-density lipoproteins [35]. Fat mass accumulates through enhanced adipocyte hyperplasia and lipogenesis, peaking by the end of the second trimester [33]. In late pregnancy, a catabolic state emerges with increased lipolysis and mobilization of fat stores [36]. These changes allow maternal tissues to rely increasingly on lipid oxidation, preserving glucose for fetal transfer [37].

#### Consequences of disrupted metabolic adaptation in pregnancy

Failure to achieve appropriate metabolic remodelling during pregnancy can unmask or exacerbate cardiometabolic dysfunction, leading to adverse maternal and fetal outcomes with long-term consequences. During pregnancy, excess adiposity compromises physiological metabolic adaptation, with impaired glucose homeostasis emerging as a central feature that predisposes to GDM. This condition is defined as hyperglycemia or glucose intolerance with onset or first recognition during pregnancy and arises from an inadequate β-cell adaptation to pregnancy-induced insulin resistance, leading to insufficient insulin supply and recurrent hyperglycemia [38]. The worldwide prevalence of GDM varies from 5% to 27%, depending on diagnostic criteria and population demographics [39]. Pre-pregnancy overweight and obesity are strongly linked to reduced insulin sensitivity that further deteriorates during gestation, thereby increasing susceptibility to GDM [40].

Chronic elevated levels of glucose, saturated lipids, and lipoproteins, as well as advanced glycation end products (AGEs) trigger a chronic, low-grade inflammation, called metainflammation [41]. Dietary AGEs and endogenous AGE production, induced by hyperglycemia, have also been linked to the development of PCOS [23]. Elevated glucose levels during pregnancy induce an inflammatory maternal-fetal environment [42], which may negatively affect the function of crucial metabolic organs like the pancreas, the adipose tissue, and the liver, thereby potentially interfering with the metabolic health of both the mother and the fetus.

Hyperglycemia in the mother leads to increased glucose levels in the fetus [43]. This condition stimulates insulin production in the fetus, increasing fat accumulation and accelerating growth, often resulting in large for gestational age infants and macrosomia [44–46]. GDM has also been associated with preterm birth, respiratory distress syndrome and hypertrophic cardiomyopathy [47, 48].

GDM is diagnosed in late second trimester by using a 75 g Oral Glucose Tolerance Test (OGTT) [49]. However, testing GDM using the traditional mid-pregnancy OGTT may result in missed opportunities for early intervention [50]. Emerging evidence suggests that first trimester detection of GDM with an early OGTT may identify at-risk pregnancies and therefore enable early interventions [51], which highlights that maternal metabolic maladaptation occur in the early stages of pregnancy.

Evidence suggest that insulin resistance, obesity and inflammation play significant roles in the development of preeclampsia, a heterogenous hypertensive pregnancy complication [52]. A potential underlying mechanism involves the shared signalling pathway of insulin and vascular endothelial growth factor, the phosphatidylinositol-3-kinase pathway, which is impaired in insulin resistance and is essential for angiogenesis and vasodilation. Consequently, it is possible that insulin resistance and hyperinsulinemia may cause endothelial dysfunction, thereby contributing to the pathogenesis of preeclampsia [53].

#### Gestational and postpartum weight gain

Maternal pre-pregnancy weight affects pregnancy development and birth outcomes. An increased pre-pregnant BMI is related with increased risk of GDM, preeclampsia, premature birth, and stillbirth among others [54]. However, excessive gestational weight gain is becoming increasingly relevant. Recent data indicate that women who gain excessive weight during pregnancy have a higher likelihood of developing preeclampsia, undergoing cesarean section, and delivering large-for-gestational-age infants, regardless of their pre-pregnancy BMI [55, 56]. Moreover, a great proportion of women with excessive gestational weight gain do not regain their pre-pregnancy weight postpartum, which doubles the risk of pregnancy and birth complications in subsequent pregnancies. For instance, the inter-pregnancy weight retention is associated with an increased risk of GDM, preeclampsia, and caesarean delivery in the second pregnancy, even among women with normal weight [57]. Consequently, implementing strategies to maintain a stable weight between pregnancies seems to be a crucial approach for reducing the risk of adverse perinatal outcomes in subsequent pregnancies.

### Metabolic (Mal-)adaptations after pregnancy in health & disease

#### Long-term health trajectories following pregnancy-related metabolic disorders

GDM usually resolves immediately postpartum, however it still poses long-term risks for both mother and child to develop NCDs [58], which are recognized as diseases associated with adult lifestyle such as hypertension, cardiovascular diseases, diabetes and obesity. According to the World Health Organization, annually they account for the 74% of deaths all over the world [59], representing a significant public health concern. It is well stablished that women who have experienced GDM face around ten times greater risk of developing type 2 diabetes (T2D) later in life compared to women without a history of GDM [60]. In GDM, it has been shown that the risk of developing T2D increases linearly over a woman’s lifetime, starting at approximately 20% ten years after pregnancy and reaching around 60% fifty years post-pregnancy [61]. These findings highlight the importance of postpartum screening to identify women at a higher risk of disease progression and to implement preventive strategies. In this context, the American Diabetes Association recommends screening women with a history of GDM between 4 and 12 weeks postpartum, followed by a long-term follow up every one to three years [62]. A comprehensive approach that includes systematic documentation, lifestyle counseling, and coordinated long-term follow-up is essential to ensure appropriate risk assessment and management of these patients.

#### Impact of maternal metabolic disease on the offspring’s lifelong metabolic health

Pregnancy is not only a window into the future health of the mother, but also into the offspring’s long-term health. The Developmental Origin of Health and Disease hypothesis emphasizes the link between maternal metabolism exposure in utero and long-term consequences for physiological function and health, such as the risk for metabolic disorders and respiratory dysfunction [63]. This transgenerational approach is based on a theory by Barker et al., postulating that an adverse environment in utero alters the metabolism and body’s structure, leading to coronary heart disease in the future [64, 65]. During gestation, fetuses are highly sensitive to alterations in the maternal nutritional and non-nutritional milieu, leading to programmed modifications in organ structure, gene expression and cellular pathways [66]. Several lines of evidence suggest that the acquisition of epigenetic changes during embryonic and fetal development may be the mechanism through which the maternal environment exerts intergenerational effects on offspring [67]. Epigenetic modifications are heritable changes in gene expression and chromatin structure that occur without altering the DNA sequence. These epigenetic modifications include DNA methylation, histone modifications, microRNAs variations and/or chromatin remodeling [66, 68]. DNA methylation demonstrates a high dynamic during embryogenesis, resulting in an establishment of methylation patterns during embryogenesis, fetal development and early postnatal life [66, 68, 69]. Histone protein modifications (methylation, ubiquitination or acetylation) result in either an activated or repressed gene expression [69] with an association to obesity, hyperlipidemia and hyperphagia, due to their role in the regulation of the principal adipogenic transcription factor [66]. Fetal programming is associated with placental dysfunction, which is mediated trough gene and environment interactions and can be seen in conditions such as intrauterine growth restriction [70]. Since metabolic dysregulation is a risk factor for multiple pregnancy complications, its impact on placental function is likely to play a central role in fetal programming. In PCOS, in utero exposure to maternal androgenemia has been linked to adverse effects on the offspring’s future health [71]. Risal et al. demonstrated that F1 generation daughters of women with PCOS are more likely to be diagnosed with PCOS. In mice, increased anogenital distance and irregular estrous cycles were observed in the F1-F3 offsprings after androgenization of F0 dams, suggesting transgenerationally altered gene expression of oocytes due to increased prenatal androgen exposure [71]. The germline, a cell line specialized in transmitting genetic material from one generation to another, may lead to the altered phenotype through the F3 generation [24, 66].

GDM is associated to pathological maternal leptin upregulation, and this upregulation can also be seen in preeclampsia [72]. Therefore, leptin may play a role in placental insufficiency and exert pro-hypertensive effects during pregnancy [73]. Growing evidence supports that epigenetic mechanisms during GDM are associated with offspring health. A study employing a two-step Mendelian randomization approach demonstrated that elevated maternal fasting glucose in the second trimester is associated with higher neonatal leptin levels, by a decrease in DNA methylation near the *LEP* gene promoter in cord blood. These results highlight a causal relation between maternal hyperglycemia and epigenetic regulation of leptin in offspring, contributing to long-term programming of excessive adiposity later in life [74]. In line with these findings, a mediation analysis revealed that increased DNA methylation of the principal adipogenic transcription factor GC1α gene locus in the placenta, responsible for glucose and lipid metabolism, was correlated with higher leptin levels in the cord blood of offspring exposed to maternal hyperglycemia in the second trimester [75]. These findings collectively suggest that epigenetic modifications induced by maternal metabolic dysregulation play a significant role in programming offspring’s metabolic and vascular health, potentially leading to increased risks of obesity and endothelial dysfunction later in life. More longitudinal human cohort studies, including the F0 until the F3 generation, are necessary to further evaluate the transgenerational inheritance of metabolic and endocrine diseases.

#### Impact of incretin-based therapies on maternal metabolic changes

**Fig. 2:**
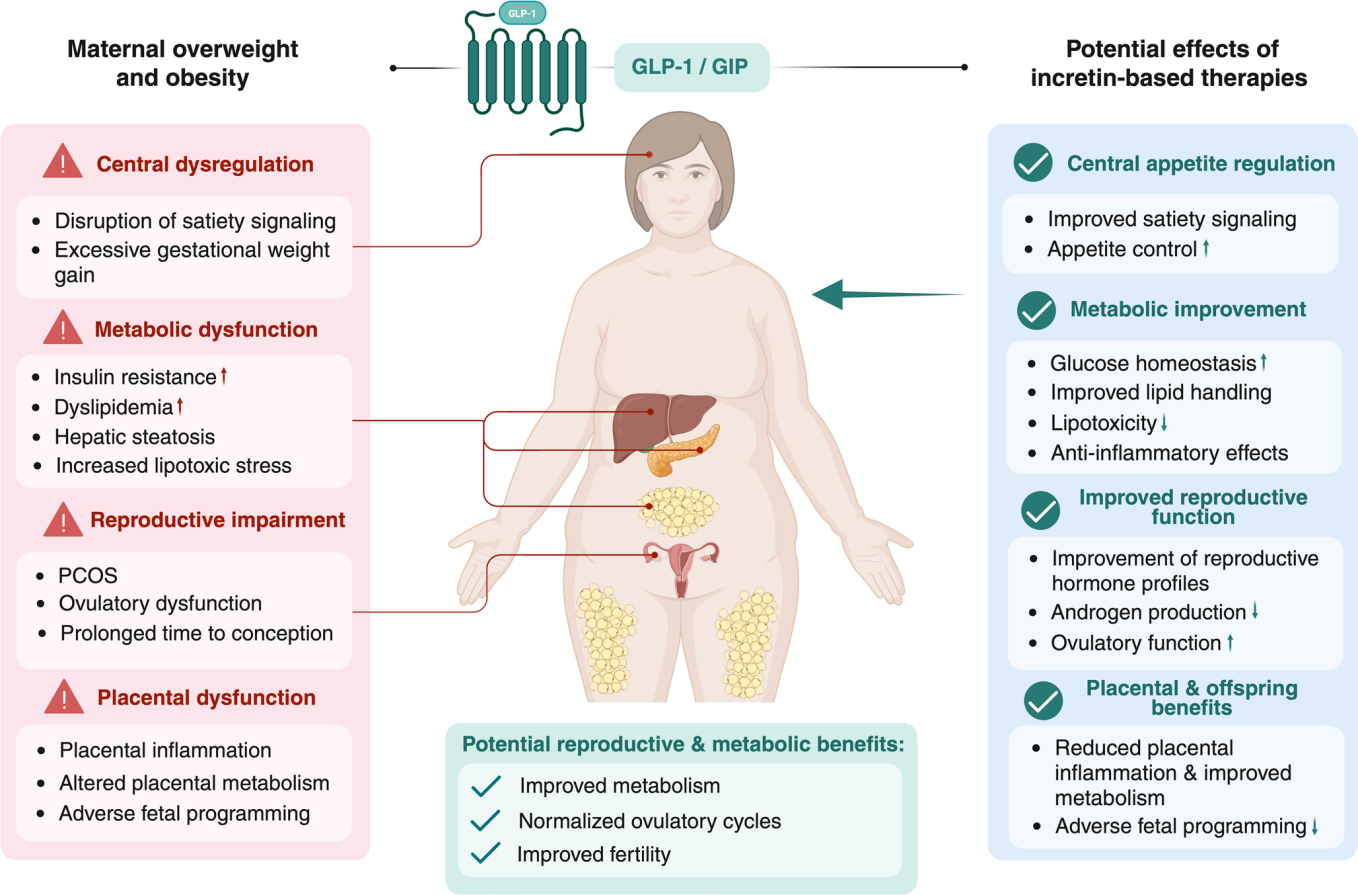
Illustrates the proposed effects of incretin-based therapies on obesity-associated maternal metabolic, reproductive, and placental dysfunction during pregnancy. GLP-1/GIP-mediated pathways may improve appetite regulation, glucose and lipid homeostasis, inflammatory balance, reproductive function and placental health, with potential benefits for maternal outcomes and fetal programming

Glucagon-like peptide-1 (GLP-1) and glucose-dependent insulinotropic polypeptide (GIP), incretin hormones, are key modulators of metabolic adaptation during pregnancy, linking nutrient sensing to pancreatic, placental, and peripheral metabolic responses. Incretin-responsive pathways support metabolic flexibility during gestation and may ameliorate maladaptation in metabolically dysregulated pregnancies (Fig. 2) [76].

Incretins are essential for maintaining glucose homeostasis in the face of progressive gestational insulin resistance. Women suffering from obesity or a history of GDM enter the pregnancy with pre-existing insulin resistance, further exacerbating the decreased insulin sensitivity that is associated with greater nutrient availability for fetal growth [77]. This superimposed insulin resistance leads to an increased insulin response, affecting placental development. Placental cytokines and hormones are increased and enhance insulin resistance in maternal tissue, promoting fetal nutrient supply and therefore leading to increased fetal growth [77, 78], which, as described in Chap. 2.3.2, increases the risk for development of adult MetS. GLP-1 signaling compensates for the decline in maternal insulin sensitivity by potentiating glucose-stimulated insulin secretion and promoting expansion of insulin-secreting β-cell mass [79]. Inadequate activation of these compensatory mechanisms contributes to GDM, underscoring the importance of intact incretin–β-cell crosstalk during pregnancy [80]. In addition to insulinotropic effects on β-cells, both GIP and GLP-1 play a role in regulating glucagon secretion of pancreatic α-cells [81]. Incretin-based therapies may improve appropriate fetal growth in pregnancies complicated by pre-existing insulin resistance, as seen in GDM or obesity, by improving insulin sensitivity as well as glycemic control [82].

GLP-1 signaling modulates the lipid shift in the catabolic state of pregnancy, by suppressing adipose tissue lipolysis, improving hepatic lipid handling, and reducing ectopic lipid accumulation [83]. Dysregulation of this axis contributes to maternal hypertriglyceridemia, hepatic steatosis, and lipotoxic stress, features commonly observed in metabolically complicated pregnancies [84]. Both GLP-1 and GIP exert indirect effects on hepatic lipid and glucose metabolism [85, 86]. GIP and GLP-1 activate cyclic adenosine monophosphate/ adenosine monophosphate (AMP)-activated protein kinase signaling in hepatocytes, promoting fatty acid β-oxidation and suppressing lipogenesis through downregulation of sterol regulatory element-binding protein 1c and fatty acid synthase [87]. By stimulating adiponectin secretion from adipose tissue, hepatic gluconeogenesis and free fatty acid accumulation are further reduced [88], thereby attenuating hepatic steatosis.

Pregnancy is associated with increased mitochondrial demand in maternal liver, adipose tissue, skeletal muscle, and placenta, requiring adjustments in oxidative phosphorylation efficiency, mitochondrial dynamics, and antioxidant defences [89]. Controlled production of reactive oxygen species supports cellular adaptation; however, excessive oxidative stress contributes to placental dysfunction and systemic inflammation [90].

GLP-1 signaling has been shown to improve mitochondrial efficiency, enhance fatty acid oxidation, and reduce oxidative stress through activation of AMP-activated protein kinase and downstream antioxidant pathways [91, 92]. These effects are particularly relevant in late gestation, when mitochondrial overload and reduction-oxidation imbalance are most pronounced.

At the maternal–fetal interface, the placenta integrates maternal metabolic cues with nutrient transport and endocrine function. Placental nutrient-sensing pathways (mechanistic target of rapamycin and AMP-activated protein kinase signaling) respond to maternal glucose, lipid, and amino acid availability, adjusting placental growth, transporter expression, and hormone secretion. GLP-1 receptors (GLP-1R) have been identified in placental tissue, suggesting that GLP-1 signaling may directly influence placental metabolism, vascular function, and inflammatory tone. Through these actions, GLP-1 may indirectly shape fetal nutrient exposure and growth trajectories, linking maternal metabolic status to developmental programming [93].

In addition, GLP-1 is produced centrally by neurons in the nucleus of the solitary tract and functions as a key neurotransmitter signaling satiety through widespread expression of GLP-1Rs in the hypothalamus and other evolutionarily conserved brain regions [94, 95]. GIP potentiates GLP-1–induced anorexigenic signaling and precursor peptide proopiomelanocortin neuron activation, resulting in additive effects on body weight regulation. During pregnancy, when maternal energy requirements increase, fine-tuning of these circuits is essential to support adaptive hyperphagia while preserving metabolic homeostasis. In the context of maternal obesity, disruption of incretin-sensitive hypothalamic circuits may impair appropriate adaptations of appetite control, contributing to excessive gestational weight gain and long-term alterations in metabolic setpoints.

Furthermore, maternal insulin resistance is associated with greater inflammation during pregnancy [96], and GLP-1 exerts anti-inflammatory effects by modulating immune cell activation, reducing pro-inflammatory cytokine release, and improving gut barrier integrity, thereby linking metabolic and immune adaptations during pregnancy [97]. Taken together, incretin-based therapy during pregnancy could potentially improve long-term health of mothers and offsprings as they could interact with mechanisms proposed in fetal programming.

## Tackling the epidemic: current strategies, interventions, and evidence

These long-term health outcomes associated with overweight and obesity underscore the need for effective therapeutic strategies. Although available interventions act through diverse molecular and functional mechanisms, they share the overarching goal of achieving clinically meaningful and sustained weight loss, ultimately aiming to reduce obesity-related metabolic and cardiovascular complications (Table 1) [98]. Lifestyle modifications represent the most accessible approach; however, they can also be the most challenging to implement or sustain over a long period of time. They typically include dietary restrictions such as low-carb or low-fat diets [99] as well as time-restricted eating [100] in combination with increased physical activity, including exercise programs or step-based goals [101]. However, in order to achieve a clinically significant benefit, structured professional behavioral counseling is recommended [102], which is often difficult to provide within primary care settings. Digital health applications, group- and telephone-delivered lifestyle interventions can support tracking, facilitate the implementation of lifestyle interventions, thereby increasing patient compliance [102]. While these first-line strategies remain the foundation of obesity management, their efficacy is highly variable [103, 104]. A substantial proportion of individuals will fail to achieve or maintain sufficient weight reduction following lifestyle adjustments, thereby warranting the use of pharmacological or surgical therapies.

**Table 1:** Overview of the discussed interventions and their current recommendations during pregnancy and the breastfeeding period

Intervention	Physiological foundation	Representatives	Indication	Mechanism	Adverse effects	Pregnancy relevance	Reference
Lifestyle therapies							
Dietary Intervention	↓ Energy intake	Low-carbohydrate, low-fat, time restricted eating	BMI$\:\ge\:$25 kg/m^2^	↓ anabolic hormones, ↓ thermogenesis and metabolic regulators, ↑ anti-inflammatory hormones, ↑ insulin sensitivity	Nutrient deficiency (if unbalanced)	Safe (individualized)	Napolealo et al. [105] Akbari et al. [99] Sundfør et al. [100]
Physical activity	↑ Energy expenditure	Structured exercise programs, step-based goals	BMI$\:\ge\:$25 kg/m^2^	↑ energy expenditure, AMPK-mediated, insulin-independent glucose uptake; preserved GLUT4 translocation; ↑ insulin sensitivity	Musculoskeletal injury, transient fatigue, rare cardiovascular events (high-risk individuals)	Safe - Moderate activity recommended (ACOG) - ↓ maternal and fetal adverse outcomes	O’Neill [106] Hall et al. [107]. ACOG committee opinion [108] Bull et al. [109]
Continuous glucose monitoring	Close monitoring of metabolic behavior to support lifestyle choices and nutrition education	Dexcom/Libre^®^ class	T1D, T2D (criteria-based), Off label: Obesity	Biofeedback → lifestyle modification Glycemic control (T2D, T1D)	Cost, skin irritation/contact dermatitis, measurement inaccuracy, anxiety due to data overload	Emerging evidence: Early-GDM monitoring trials ongoing; routine use not currently recommended.	Hegedus et al. [110] Asarani et al. [111] Klonoff et al. [112] Valent et al. [113]
Pharmacological therapies							
Incretin-based therapies	Regulate energy intake through hormone signaling	See Table 2.	T2D, BMI$\:\ge\:$30 kg/m^2^ or BMI$\:\ge\:$27 + weight-related comorbidity	GLP-1 receptor → cAMP ↑ → insulin ↑ / glucagon ↓ → appetite ↓, glucose ↓	GI intolerance (altered gastric emptying, emesis, constipation)	Use not recommended during pregnancy Discontinue ≥ 2 months before planned pregnancy *Ongoing evaluation on safety data*	Drucker [114] Yoo et al. [115] Zhang et al. [116] U.S. FDA [117]
Centrally acting combination therapies	Targeting hunger signaling/ appetite regulation	Naltrexone–bupropion (e.g. Contrave^®^)	BMI$\:\ge\:$30 kg/m^2^ or BMI$\:\ge\:$27 + weight-related comorbidity	Bupropion ↑ POMC + naltrexone ↓ auto-inhibition → ↓ appetite	Nausea, constipation, neuropsychiatric effects, palpitations, ↑ blood pressure	Use not recommended during pregnancy or breastfeeding Discontinue ≥2 weeks before conception or immediately if pregnant.	Apovian et al. [118] Nissen et al. [119] Duah et al. [120] Sherman et al. [121]
Peripherally acting therapies	Reduction of intestinal fat absorption	Orlistat (Xenical^®^)	BMI$\:\ge\:$30 kg/m^2^ or BMI$\:\ge\:$28 + weight-related comorbidity	Lipstatin derivative → inhibits gastric & pancreatic lipases → ↓ triglyceride hydrolysis → ↓ fat absorption → ↓ energy intake	Steatorrhea, frequent bowel movements, deficiency (Vitamin A, D, E and K), severe liver injury	Routine use not recommended during pregnancy	Duah et al. [120] U.S. FDA [122] Hauptman et al. [123] Sjostrom et al. [124] Melia et al. [125] Ballinger et al. [126]
Metabolic therapies	Improvement of insulin sensitivity	Biguanides (Metformin)	T2D Off-label use: PCOS, Obesity	↓ Gluconeogenesis → ↓ glucose Modulates gut microbiota → metabolic benefit AMPK-dependent/independent anti-inflammatory effects↑ GDF15 → weight loss (glucose lowering independent)	GI intolerance (nausea, emesis, diarrhea), Vitamin B12 deficiency	Not routinely recommended; off-label use may be considered after individual risk–benefit assessment	Foretz et al. [127] Hurley-Kim et al. [128] Toft et al. [129]
Operative (bariatric) therapies							
Combined gastric and intestinal surgery	Modification of the gastrointestinal anatomy, malabsorption	Roux-en-Y gastric bypass, one-anastomosis gastric bypass	BMI *≥*35 kg/m^2^, BMI *≥* 30 kg/m^2^ + inadequately controlled T2D	↑ Incretin secretion, ↑ Insulin sensitivity, ↑ weight loss, ↑ bile acids Restriction + malabsorption; altered gut hormone signalling	Dumping syndrome, micronutrient deficiency, anastomotic insufficiency	Safe: Delay pregnancy ≥ 12–24 months after bariatric surgery to reduce adverse outcomes	Drucker [114] Jacobsen et al. [130] Peterli et al. [131] Sillakivi et al. [132] Morgan et al. [133] Milone et al. [134] Gustavsson et al. [135] Eisenberg et al. [136]
Gastric surgery	Restriction of gastric volume	Sleeve gastrectomy, adjustable gastric banding	BMI *≥*35 kg/m^2^, BMI *≥* 30 kg/m^2^ + inadequately controlled T2D	Altered gastric emptying and satiety pathways Sleeve gastrectomy: ↑ Incretin secretion	Nutrient deficiencies, surgical complications	Safe: Delay pregnancy ≥ 12–24 months after bariatric surgery to reduce adverse outcomes	Drucker [114] Shawe et al. [137] Eisenberg et al. [136]
Endoscopy	Restriction of gastric volume	Intragastric balloons, endoscopic sleeve gastroplasty	BMI 30 kg/m^2^ − 40 kg/m^2^	Mechanical gastric restriction, delayed gastric emptying	Nausea, vomiting, rare perforation	Safe: Delay pregnancy ≥ 12–24 months after bariatric surgery to reduce adverse outcomes	Sullivan et al. [138] Perdomo et al. [139] Baratte et al. [140]

Abbreviations: *T1D* Type one diabetes, *T2D* Type two diabetes, *BMI* Body mass index, *GLP-1* Glucagon-like peptide-1, *AMPK* AMP-activated protein kinase, *POMC* Proopiomelanocortin, *GIP* Glucose-dependent insulinotropic polypeptide, *GDF15* Growth differentiation factor 15

For patients where substantial weight loss (BMI ≥ 40 kg/m^2^ or BMI ≥ 35 kg/m^2^ with at least one obesity-related complication) is needed, the use of surgical interventions is recommended [141]. Multiple studies have proven the mid to long-term (over ten years) weight loss effects of bariatric surgeries, which were associated with an overall reduction in mortality rates compared to control subjects [142–144]. However, bariatric surgery must be embedded in a structured lifestyle intervention, as long-term weight maintenance and metabolic benefit critically depend on sustained behavioral, nutritional and physical activity changes [145, 146]. Most used surgical procedures are Roux-en-Y gastric bypass and sleeve gastrectomy [147]. A broad classification can be made based on whether the intervention targets the stomach alone or both the stomach and intestine. While the primary goal of gastric surgeries (like sleeve gastrectomy) is the reduction of gastric volume through partial gastric resection, depending on the procedure, secondary effects include alterations in gut hormone secretion, in particular ghrelin, partly attributable to the removal of hormone-producing regions of the stomach [131, 132]. More recently, studies have shown that the co-expressed gut hormones GLP-1 and peptide YY are postprandially elevated in individuals following Roux-en-Y gastric bypass and vertical sleeve gastrectomy, highlighting a potential key hormonal mechanism underlying the metabolic benefits of bariatric surgery [148]. While combined gastric and intestinal surgeries also lead to reduced gastric volume, they also cause malabsorption due to the delayed contact between food and digestive juices and are associated with an increased risk of developing dumping syndrome [149, 150]. Today, complication and reoperation rates are low, reflecting advances in surgical techniques, improved patient selection and structured perioperative care [144]. Although minimally invasive approaches reduce surgical risk, bariatric procedures remain associated with complication rates of 10%–17% and reoperation rates near 7%, while perioperative mortality is comparatively low (0.08%–0.35%) [151].

Despite the promising and sustaining long-term outcomes of bariatric surgeries for patients with severe obesity, less invasive techniques should not be neglected [145, 152]. A minimally invasive option for achieving weight loss is through endoscopic bariatric therapy, which, although being less effective than surgical approaches, is often used to induce initial weight loss prior to bariatric surgery. Like gastric-targeted bariatric surgeries, endoscopic methods can achieve a gastric volume reduction without altering gastrointestinal anatomy (reviewed in [138, 139]). To minimize risks, it is recommended to postpone pregnancy until at least 12 to 24 months after bariatric surgery to reduce the risk of adverse pregnancy outcomes, including small-for-gestational-age infants and stillbirth [133–153].

In contrast, pharmacological weight-loss interventions are more readily accessible; however, there is currently insufficient evidence to define a standardized recommendation regarding preconception discontinuation, resulting in highly variable guidance depending on the specific agent [154]. These anti-obesity medications are often delivered in primary care settings where tightly regulated preconceptional protocols can often not be guaranteed. Pregnancy intention can strongly motivate preconception weight loss. Therefore, counseling should be embedded at treatment initiation [155]. The number of available pharmacological agents has increased rapidly over recent years, expanding the availability of highly effective therapeutic options to healthcare professionals, thus enabling more personalized treatment strategies [156]. Anti-obesity medications are commonly suggested for individuals with a BMI ≥ 27 kg/m^2^ in the presence of one or more co-morbidities or in patients with obesity. Several anti-obesity medications have been approved by the Food and Drug Administration (FDA) and the European Medicines Agency (EMA) and can be classified into three major groups [156]. These include peripherally acting agents like Orlistat, centrally acting combination therapies like Naltrexone–Bupropion, and, more recently, incretin-based therapies, with a particular focus on GLP-1 receptor agonists (GLP-1 RAs) and dual incretin agonists [118, 119]. Low-dose Orlistat (60 mg) is currently the only non-prescriptive anti-obesity medication approved by the FDA and EMA. It is a derivative of lipstatin and targets the activity of pancreatic and gastric lipases, which inhibits the hydrolysis of triglycerides into free fatty acids and thereby reduces their intestinal absorption, which lowers total energy intake [123]. This can lead to multiple downsides, including gastrointestinal complications like steatorrhea, or an insufficient absorption of fat-soluble vitamins [124, 125]. Centrally acting combination therapies, such as naltrexone–bupropion, target central hunger signalling pathways. Bupropion, commonly employed as an antidepressant, increases dopamine and noradrenaline levels in the brain, leading to appetite suppression, while naltrexone prolongs this effect by blocking opioid receptor–mediated feedback from beta-endorphin that would otherwise attenuate the pathway. In a double-blind, placebo-controlled study, naltrexone–bupropion was associated with significantly greater weight loss than placebo, combined with improvements in cardiometabolic risk factors [118]. However, a modest increase in blood pressure and heart rate was observed in addition to side effects, including nausea [118, 157, 158]. In this context, naltrexone–bupropion is generally not recommended as a weight loss agent for pregnant or breastfeeding women.

In recent years, multiple hormone-based pharmacological therapies for the treatment of overweight and obesity have been approved by the FDA and EMA [159]. Several of these belong to the class of incretin-based therapies (GLP-1 RAs) or dual incretin agonists (Table 2). Through activation of GLP-1R signalling, these agents influence multiple metabolic and weight-regulating pathways, including improvement of blood glucose levels, appetite regulation, and delayed gastric emptying. Consequently, GLP-1-RAs have been shown to promote cell proliferation, exert anti-inflammatory effects, improve cardiovascular health, and may help to preserve neurological functions [160]. And while their effects are mostly beneficial for patients suffering from overweight or obesity, negative side effects can include gastrointestinal or gallbladder disorders [161]. In addition, their benefits are generally limited to the duration of treatment [161]. Without further education or guidance on sustained weight control, discontinuation often leads to rapid weight regain reversal of beneficial effects on cardiometabolic indices [162].

**Table 2:** Overview of selected incretin-based therapies, their approved indications, dosing regimens, and reported weight-loss effects

Name	Indication	Maintenance dose^b^	Weight loss effect	Reference
Glucagon-like peptide-1 (GLP-1) receptor agonists				
Dulaglutide	T2D	TRULICITY^®^: 1.5 mg (if additional glycemic control is needed: 3 mg or 4.5 mg), once weekly	Not approved for weight-loss management	U.S. FDA [163]
Exenatide extended-release	T2D	BYDUREON^®^: 2 mg, once weekly	Not approved for weight-loss management	U.S. FDA [164]
Liraglutide	T2D (VICTOZA^®^), Obesity, Overweight + weight-related comorbidity^a^ (SAXENDA^®^)	VICTOZA^®^: 1.2 mg (if additional glycemic control is needed: 1.8 mg), once daily; SAXENDA^®^: 3 mg, once daily	-6.4% (mean percentage weight change from baseline to 68 weeks)^c^	U.S. FDA [165] U.S. FDA [166] Rubino et al. [167]
Lixisenatide	T2D	ADLYXIN^®^: 20 µg, once daily	Not approved for weight-loss management	U.S. FDA [168]
Semaglutide	T2D (OZEMPIC^®^), BMI$\:\ge\:$30 kg/m^2^ or BMI$\:\ge\:$27 + weight-related comorbidity^a^ (WEGOVY^®^)	OZEMPIC^®^: 0.5 mg (if additional glycemic control is needed: 1 mg/ 2 mg) once weekly; WEGOVY^®^: 2.4 mg, once weekly	-15.8% (mean percentage weight change from baseline to 68 weeks)^c^	U.S. FDA [169] U.S. FDA [159] Rubino et al. [167]
Glucose-dependent insulinotropic polypeptide (GIP) receptor and GLP-1 receptor agonist				
Tirzepatide	T2D (MOUNJARO™), BMI$\:\ge\:$30 kg/m^2^ or BMI$\:\ge\:$27 + weight-related comorbidity^a^ (ZEPBOUND^®^)	MOUNJARO™: 5 mg (if additional glycemic control is needed: maximum dosage of 15 mg), once weekly ZEPBOUND^®^: 5 mg, 10 mg, 15 mg, once weekly	-15% (5 mg), -19.5% (10 mg), -20.9% (15 mg) (mean percentage weight change from baseline to 72 weeks)^c^	U.S. FDA [170] U.S. FDA [171] Jastreboff et al. [172]

^a^Weight-related comorbid condition (e.g., hypertension, dyslipidemia)

^b^Route of administration: subcutaneous injection for all listed drug

^c^As an adjunct to lifestyle intervention (diet and physical activity)

Abbreviations: *T1D* Type one diabetes, *T2D* Type two diabetes, *BMI* Body mass index

## Incretin-based therapies in reproduction

### Potential role of incretin-based therapies in improving reproductive function

As previously mentioned, in women, excess adiposity and metabolic dysregulation disrupt hypothalamic-pituitary-gonadal axis function, leading to altered gonadotropin secretion, impaired folliculogenesis, ovulatory dysfunction, and menstrual irregularities. Hyperinsulinemia further exacerbates this endocrine imbalance by directly stimulating ovarian theca cells, increasing androgen production, and promoting the hormonal milieu characteristic of PCOS, a leading cause of anovulatory infertility [173, 174].

Incretin-based therapies have transformed the management of obesity and T2D by inducing robust and sustained improvements in glycemic control and body weight [175]. From a reproductive standpoint, the systemic effects of incretin-based therapies directly target central mechanisms underlying obesity-associated infertility. In particular, reductions in hyperinsulinemia may restore ovarian steroidogenesis, mitigate androgen excess, and promote the resumption of ovulatory cycles, especially in women with PCOS or obesity-related anovulation [173, 176]. Preclinical evidence supports these concepts: GLP-1 RAs attenuate ovarian inflammation and endocrine dysfunction in a mouse model of PCOS, leading to improved metabolic parameters, normalized estrous cyclicity, restored ovarian morphology, and balanced sex hormone profiles via activation of the AMP-activated protein kinase/ Sirtuin 1/ NF-κB signaling pathway [176].

Mechanistically, accumulating evidence indicates that female reproductive tissues express multiple gut hormone receptors, including those for GLP-1, GIP, and peptide YY, suggesting a direct role for these hormones in the regulation of reproductive function and hormone secretion [177–179]. Consistent with this, GLP-1–based multi-agonists improve both metabolic and reproductive phenotypes in two distinct mouse models of PCOS. Notably, a GLP-1/estrogen co-agonist demonstrated superior efficacy compared with other combinations (GLP-1/GIP and GLP-1/GIP/glucagon) as well as metformin, normalizing metabolic parameters and ovarian cyclicity through central hypothalamic mechanisms without eliciting uterotrophic estrogenic effects [180]. Recent studies further reveal that these receptors are expressed in both ovarian and adrenal tissues, with their expression dynamically altered in metabolic disorders such as obesity and diabetes, highlighting a potential mechanistic link between gut hormones, metabolic state, and reproductive regulation [181]. However, their physiological relevance in humans remains poorly defined, clinical data are beginning to emerge. In a randomized, placebo-controlled trial, liraglutide in a daily dosage of 3 mg significantly reduced body weight and androgen excess compared with placebo in women with obesity and PCOS, with an acceptable safety profile primarily characterized by mild to moderate gastrointestinal side effects [182].

Collectively, these findings suggest that incretin-based therapies act on upstream metabolic and inflammatory pathways that impair reproductive function, highlighting them as promising disease-modifying strategies in metabolically driven infertility. However, larger, well-designed prospective studies are required to fully establish their reproductive safety and therapeutic efficacy.

### Distinct reproductive impacts of modern obesity therapies

Pre-pregnancy weight loss represents a key intervention to improve reproductive outcomes. Even modest weight reductions of 5%-10% can enhance fertility in women suffering from obesity and anovulatory infertility, while simultaneously lowering the risk of pregnancy complications such as preeclampsia, GDM, preterm birth, macrosomia, and stillbirth [183, 184]. By improving insulin sensitivity, reducing hyperandrogenism, and restoring ovulatory function, incretin-based therapies may rapidly enhance fertility in women with obesity, introducing the potential for unintended conception if contraception is not appropriately used. On the one hand, the dual GLP-1 and GIP receptor agonist tirzepatide is more effective than GLP-1 RAs due to a greater delay on gastric emptying. On the other hand, this leads to a greater impact on the absorption of oral hormonal contraceptives in comparison to other GLP-1 RAs [185]. Therefore, it is recommended that patients using oral hormonal contraceptives switch to a non-oral contraceptive method, or add a barrier method of contraception, for four weeks after initiation with tirzepatide and for four weeks after each dose escalation [170]. Crucially, the effects of incretin-based therapies during early gestation remain poorly characterized. Potential effects on gestational weight gain, oocyte quality, and epigenetic modifications remain poorly understood, despite the increasing use of these agents to treat obesity, with fertility improvement often arising as a secondary consequence. Addressing these knowledge gaps through carefully designed studies is essential to guide safe, evidence-based reproductive care in this growing patient population.

### Comparison of Incretin-based therapies and metformin in PCOS

Metformin has traditionally been used to improve metabolic and reproductive outcomes in patients suffering from PCOS, however recent evidence suggests that incretin-based therapies, particularly GLP-1 RAs may provide superior benefits. Indeed, meta-analyses of randomized controlled trials indicate that GLP-1 RAs more effectively enhance insulin sensitivity, reduce BMI and abdominal girth, and improve reproductive hormone profiles, including increases in FSH and sex hormone-binding globulin and reductions in total testosterone and free androgen index [186–189]. In women suffering from PCOS and overweight or obesity, a combination therapy with the GLP-1 RA semaglutide and metformin significantly improves body weight reduction, insulin resistance, menstrual irregularities and natural pregnancy rates, compared to metformin monotherapy [190]. In terms of tolerability, GLP-1 RAs are most commonly associated with mild gastrointestinal effects such as nausea and headache, whereas metformin is more frequently linked to diarrhea [187]. Collectively, these findings support the use of incretin-based therapies either alone or in combination with metformin as a promising approach for managing PCOS, particularly in insulin-resistant women with obesity, although the evidence remains of moderate quality and underscores the need for further high-quality trials to establish long-term efficacy and safety.

## Safety and use of incretin-based therapies during pregnancy

A continuous treatment with incretin-based medication, such as tirzepatide, is necessary to maintain and augment initial weight loss in women with obesity [191]. However, recommendations suggest to not use incretin based-therapies during pregnancy and to discontinue medication one to three months before pregnancy, depending on the particular drug [192] Therefore, the cessation of GLP-1 RAs before conception or in early pregnancy is associated with excessive gestational weight gain and a higher risk of adverse pregnancy outcomes, such as GDM, hypertensive disorders of pregnancy and preterm birth [193]. Given the widespread use of incretin-based agents in women planning pregnancy, inadvertent administration in early pregnancy is, in some cases, inevitable. The effects of peri-pregnancy incretin-based medication on fetal health remains ambivalent, as several studies showed encouraging as well as concerning results. Recent rodent studies associated GLP-1 exposure to increased fetal mortality, reduced fetal weight and fetal growth restriction [93, 194]. Observational studies did not find a greater risk of malformations or major birth defects associated with periconceptional exposure to GLP-1 RAs compared to insulin treatment in pregnant women with T2D, respectively [195, 196]. In pregnant women with pre-gestational diabetes, GLP-1 RA use at some point during pregnancy was not associated with an increased overall rate of any congenial malformation, but a possible increase in genital and urinary malformations [197]. Moreover, studies investigating the vitamin and micronutrient supply under GLP-1 RA and dual-agonist therapy are limited. The decision for continuing GLP-1 RA until pregnancy confirmation vs. discontinuing them prematurely is a balancing process between risking the reversal of weight loss benefits and potential harmful effects on the offspring [198], highlighting the need to identify patients that could particularly benefit from the use of incretin-based therapies during pregnancy. Incretin-based therapies improve glycemic control in obese individuals with T2D [169, 170], and may also be an alternative approach to treat GDM. In patients with GDM, insulin is considered the preferred treatment. Metformin is a reasonable alternative choice with limited use of other medication [199]. Currently, a pilot pregnancy study investigating the pharmacokinetics and pharmacodynamics of the already decommissioned drug for T2D, exenatide, an incretin mimetic, in women with GDM is under way [200]. Studies with long-term follow-up are needed to investigate the placental transfer of incretin-based therapies and their long-term effects on exposed offsprings.

## GLP-1 agonists after pregnancy

### Postpartum metabolic health: window of opportunity to improve maternal health

The postpartum period offers a window of opportunity to tackle but also prevent metabolic disorders in women. Pre-pregnancy obesity in women is associated with higher postpartum weight gain [201], highlighting the need for weight-loss interventions, as postpartum weight retention is associated with glucose intolerance and the progressive development of an adverse vascular risk factor profile during the first five years after delivery [202]. Moreover, weight reduction between pregnancies lowers the risk of hypertensive complications, stillbirth and fetal macrosomia in future pregnancies [203]. Maternal obesity following pregnancy is associated with a higher risk for NCDs not only for the mother but also for their children [203], emphasizing the need for effective postpartum weight loss interventions. A healthy diet and exercise are substantial parts of postpartum weight loss management, though for many women, these approaches are shown to be unsuccessful [204]. A recent registry-based study conducted in Denmark identified an increased postpartum use of GLP-1 RAs since their introduction for weight loss in late 2022 [205], suggesting its emerging role in the management of postpartum obesity. Tirzepatide shows improvements in cardiovascular and metabolic risk factors, such as waist circumference, systolic and diastolic blood pressure, and fasting insulin, lipid, and aspartate aminotransferase levels in a cohort of patients suffering from obesity [172], emphasizing the potential role of incretin-based therapies to improve the long-term women’s health.

The effect of incretin-based therapies on lactation and breastfeeding remains uncertain. The drug transfer of subcutaneous GLP-1 RA semaglutide into human milk was investigated in a small cohort consisting of eight women [206]. Semaglutide was not detected in the collected samples, indicating that its transfer to human milk is low and unlikely to be absorbed by the offspring, as the protein is degraded in the infant’s gastrointestinal system [204, 206, 207]. Recent rodent studies propose a systemic effect of GLP-1 RA liraglutide exposure during lactation on offsprings, as neonatal growth was reduced [207]. Another concern regarding the use of incretin-based medication after pregnancy addresses the induced accelerated weight loss and initial malnutrition state, which may result in decreased milk production and altered milk composition [204, 206]. Therefore, women should be advised to meet their daily nutrient recommendations [206]. Collectively, more high-quality studies are necessary to evaluate the safety of incretin-based therapies during lactation and breastfeeding.

### Impact on long-term fetal trajectories and child development

Maternal circulating GLP-1 levels progressively decline throughout pregnancy in mice. Here, the administration of the GLP-1 RA semaglutide lead to fetal body weight reduction and alterations in placental development and placental vascular structure, suggesting that the downregulation of GLP-1 during pregnancy plays a crucial role for placental and fetal growth [194]. Human ex vivo placenta explant experiments demonstrated that dulaglutide, a long-acting GLP-1 RA, crosses the placental barrier at term, while not being able to cross the cytotrophoblast layer, indicating that dulaglutide transport across the placenta is neonatal Fc receptor-mediated. However, during the early stages of pregnancy, where Fc-mediated transfer is expected to be absent or minimal, dulaglutide showed very low permeability in BeWo cell in vitro experiments, which may particularly be relevant in cases of unexpected early-pregnancy exposure to GLP-1 RAs due to unplanned pregnancies under these medications [208]. Moreover, Kuoni et al. suggested that dulaglutide does not impair endocrine pathways crucial for maintaining pregnancy, such as the secretion of leptin and human chorionic gonadotropin as well as glucose and lactate metabolism [208]. Maternal weight loss prior to pregnancy in mothers with obesity may not only improve maternal and offspring health, but also placental inflammation, oxidative stress, and dysregulated metabolism, that are caused by maternal obesity. A recent mouse study by Rodrigo et al. demonstrated that preconceptional liraglutide treatment improved certain metabolic aspects of placental health; however, it did not reduce markers of oxidative stress or inflammation. In contrast, dietary modification initiated before pregnancy and maintained throughout gestation significantly decreased both oxidative stress and inflammatory markers. The lack of effect of liraglutide on these parameters may be related to the substantial gestational weight gain observed following discontinuation of GLP-1 RAs during pregnancy [209]. Beyond maternal metabolic effects, GLP-1 RAs may also influence fetal development through alterations in gene expression. Ramamoorthy et al. reported upregulation of genes linked to skin abnormalities, neurodevelopmental disruption, liver dysfunction, and pulmonary pathology in the offspring of mice exposed to recombinant GLP-1 during gestation, raising concerns about potential adverse long-term effects on offspring health [93]. Taken together, high-quality, long-term follow up studies are necessary to evaluate the effects of incretin-based therapies on long-term fetal trajectories and child development.

### Personalized medicine approaches in incretin-based therapies

Incretin-based therapies have revolutionized the management of overweight and obesity [210]. However, there are individual responses to incretin-based therapies regarding weight loss, which are based on multiple factors, such as desensitisation and genetics. Jastreboff et al. demonstrated that 31,5% of patients treated with 5 mg tirzepatide s.c./weekly and 21,9% of patients treated with 10 mg tirzepatide s.c./weekly, did not reach a weight reduction of 10% or more at week 72 [172]. This highlights the need for personalized obesity management, targeting the interindividual mechanisms that lead to obesity [211].

One promising approach to optimize pharmacotherapy-based outcomes is the identification of distinct obesity phenotypes. Acosta et al. identified different obesity phenotypes and proposed a framework for selection of anti-obesity medications based on these phenotypes [210, 212]. In this real-world-study, phenotype-based selection of anti-obesity medications resulted in a 1.75- fold greater weight loss after one year compared to a standard of care approach [191], emphasizing the importance of personalized therapies to reduce heterogeneity in weight loss outcomes. Patients with an abnormal postprandial phenotype (decreased duration of fullness, objectively quantified by rapid gastric emptying) could especially benefit from GLP1-RA therapy, as these medications delays gastric emptying and may restore satiety mechanisms [210]. However, widespread implementation of obesity phenotyping in routine clinical practice remains challenging due to time constraints and cost considerations [210].

Beyond its therapeutic role, GLP-1 may also serve as a biomarker to guide individualized treatment strategies. Measurement of circulating GLP-1 levels could provide insight into mechanisms of metabolic dysregulation, particularly with respect to insulin secretion and glycemic control [213]. Future studies integrating genetic, epigenetic, microbiome, and environmental factors will be essential to further advance personalized medicine approaches in obesity treatment.

### Practical reommendations for clinicans

Before concluding, we wish to highlight several practical recommendations for clinicians managing obesity in women of reproductive age, synthesized from the evidence discussed throughout this review.

#### Counsel at treatment initiation

Given that incretin-based therapies may accelerate fertility restoration, contraceptive counseling and pregnancy intention should be addressed at the start of treatment in all women of reproductive age.

#### Plan preconception discontinuation

Current guidance recommends discontinuing GLP-1 receptor agonists one to three months before planned conception, depending on the agent. Clinicians should proactively establish a discontinuation plan in women actively trying to conceive.

#### Monitor for unplanned pregnancy

As inadvertent first-trimester exposure is increasingly common, clinicians should counsel patients to perform early pregnancy testing and seek a prompt medical appointment if pregnancy occurs while on therapy.

#### Address post-discontinuation weight regain

Cessation of incretin-based therapy is frequently associated with rebound weight gain. Adjunct lifestyle interventions like dietary optimization and physical activity should be actively supported to mitigate this risk.

#### Exercise caution in the postpartum period

Data on GLP-1 RA transfer into breast milk remain limited. Until robust evidence is available, these agents should be used with caution during lactation, and patients should be advised to maintain adequate nutritional intake.

#### Consider bariatric surgery timing carefully

Women who undergo bariatric surgery should be advised to delay conception for at least 12–18 months postoperatively, and to ensure close nutritional monitoring during pregnancy.

Given the current gaps in evidence, clinicians are encouraged to engage in shared decision-making with their patients, balancing the well-established benefits of weight loss against the still-evolving understanding of treatment safety across the reproductive lifespan.

## Conclusion

Obesity has reached pandemic proportions, significantly disrupting women’s reproductive health, with lifelong consequences for the mother and offsprings. Among women of reproductive age, incretin-based therapies are increasingly used to induce clinically meaningful weight loss. Besides reducing weight and potentially improving metabolically driven infertility, incretin-based therapies may also ameliorate metabolic dysregulation during pregnancy through various mechanisms, including effects on insulin regulation, lipid and placental metabolism, as well as anti-oxidative and anti-inflammatory pathways, for example in pregnancies complicated by GDM. As incretin-based therapies are currently not approved for use during pregnancy, their discontinuation often leads to excessive maternal weight regain. This rebound weight gain may increase the risk of adverse outcomes, including the development of preeclampsia, thereby posing significant risks to both the mother and the fetus. Moreover, incretin-based therapies may accelerate fertility, increasing the likelihood of rapid or unplanned pregnancies, while bariatric surgery, another intervention to achieve and maintain sufficient weight reduction, often necessitates postponement of conception to reduce maternal-fetal risks. Collectively, rigorous research into the long-term efficacy and safety of incretin-based therapies in the preconceptional, pregnancy, and postpartum periods-together with the development of personalized therapeutic strategies for obesity holds transformative potential not only to optimize individual maternal health, but also to disrupt the transgenerational transmission of obesity and metabolic disease shaping healthier outcomes for future generations.

## Data Availability

No datasets were generated or analysed during the current study.

## Abbreviations

AGEs: Advanced glycation end products
AMP: Adenosine monophosphate
BMI: Body mass index
EMA: European Medicines Agency
FDA: Food and Drug Administration
FSH: Follicle-stimulating hormone
GDM: Gestational diabetes mellitus
GIP: Glucose-dependent insulinotropic polypeptide
GLP-1: Glucagon-like peptide-1
GLP-1R: Glucagon-like peptide-1 receptor
GLP-1 RA: Glucagon-like peptide-1 receptor agonist
LH: Luteinizing hormone
MetS: Metabolic syndrome
NCDs: Non-communicable diseases
OGTT: Oral Glucose Tolerance Test
PCOS: Polycystic ovarian syndrome
T2D: Type two diabetes
